# Novel Inulin Derivatives Modified with Schiff Bases: Synthesis, Characterization, and Antifungal Activity

**DOI:** 10.3390/polym11060998

**Published:** 2019-06-04

**Authors:** Yuan Chen, Yingqi Mi, Xueqi Sun, Jingjing Zhang, Qing Li, Naiyun Ji, Zhanyong Guo

**Affiliations:** 1 Key Laboratory of Coastal Biology and Bioresource Utilization, Yantai Institute of Coastal Zone Research, Chinese Academy of Sciences, Yantai 264003, China; yuanchen@yic.ac.cn (Y.C.); yqmi@yic.ac.cn (Y.M.); xqsun@yic.ac.cn (X.S.); jingjingzhang@yic.ac.cn (J.Z.); qli@yic.ac.cn (Q.L.); 2 Center for Ocean Mega-Science, Chinese Academy of Sciences, 7 Nanhai Road, Qingdao, 266071, China; 3 University of Chinese Academy of Sciences, Beijing 100049, China

**Keywords:** inulin, Schiff base, antifungal activity

## Abstract

In this paper, we report chemical modifications of inulin by seven kinds of aromatic Schiff bases, which are different from their substituent groups. The obtained inulin derivatives were confirmed by FTIR, ^1^H NMR, and ^13^C NMR. Then, we studied their antifungal activity against four kinds of plant pathogens involving *Botrytis cinerea, Fusarium oxysporum* f. sp. cucumerium Owen, *Fusarium oxysporum* f. sp. niveum, and *Phomopsis asparagi* by the mycelium growth rate method. The results revealed that all inulin derivatives were endowed with significant antifungal activity compared to inulin. Among them, 6-amino-(*N*-4-chlorobenzylidene)-6-deoxy-3,4-di-O-acetyl inulin (4CBSAIL) and 6-amino-(*N*-3,4-dichlorobenzylidene)-6-deoxy-3,4-di-O-acetyl inulin (3,4DCBSAIL), which were synthesized from p-chlorobenzaldehyde and 3,4-dichlorobenzaldehyde, could completely inhibit the growth of the test fungi at 1.0 mg/mL. The inhibitory indices of the inulin derivatives were related to the type, position, and number of substituent groups (halogens) on the Schiff bases. The results confirmed that it was feasible to chemically modify inulin with Schiff bases to confer high antifungal activity to inulin. The products described in this paper have great potential as alternatives to some harmful pesticides used for plant disease control.

## 1. Introduction

There is considerable interest in developing polysaccharides for biological applications because they are cheap, nontoxic, biocompatible, and biodegradable. Inulin is one such kind of plant-derived polysaccharide and is distributed in more than 36,000 kinds of vegetables, as well as some microorganisms [1]. The most abundant sources of inulin are the tubers of *Helianthus tuberosus* and *Cichorium intybus* [2]. Inulin is made up of _D_-fructofuranose units, which are linked by β-_2,1_-glycosidic bonds, and usually combined with a glucose residue at the terminal [3]. It is worth mentioning that this particular structure makes inulin very popular as a kind of sugar or fat replacer in the food industry because inulin cannot be digested in the small intestine and thus has few calories [4,5]. Moreover, inulin has been extensively developed in pharmaceutical applications as a drug carrier to cure colon diseases since it avoids decomposition by digestive enzymes [6,7,8,9,10]. Also, as a kind of prebiotic, inulin could promote the proliferation of some beneficial bacteria in the colon, mainly including *Bifidobacteria* and *Lactobacilli*, thus inhibiting the growth of harmful bacteria and enhancing the intestinal microbial balance to stimulate the immune system [11]. Not only that, inulin could also promote the body’s absorption of minerals, prevent colon cancer, and relieve constipation, giving inulin the reputation as a physiologically functional food [12,13,14,15]. In recent decades, inulin has found some new applications in fields such as drug carriers, effluent treatments, biofuels, tissue engineering scaffolds, and so forth. [16,17,18,19,20]. However, most inulin applications are direct additions of its raw material, and considering its many admirable physiological functions and natural abundance, especially compared with well-developed polysaccharides such as chitosan and cellulose, the utilization of inulin is obviously insufficient.

In order to develop polysaccharides, chemical modification is regarded as a targeted, convenient, and highly efficient strategy that could improve and even introduce new characteristics for specific applications. In recent years, inulin modification for industrial applications has received more attention [21,22]. However, despite inulin having many natural advantages, its deficiency in some biological activities, such as antimicrobial activity, is an important factor which limits its applications [23,24]. As a consequence, some efforts based on chemical modification have been carried out to enhance inulin’s biological activity [25,26]. Schiff bases, characterized by the –N=CH– (imine) group, are of considerable interest in many fields such as fungicides, catalysts, and preservatives [27,28,29,30]. In the last few decades, Schiff bases have been a popular topic of study for structural modifications of polysaccharides due to their expanded biofunctional properties [31,32,33]. However, a Schiff base modifying inulin has been rarely reported in the literature.

It has been reported that the iminophosphoranes generated by Staudinger reduction of 6-azido-6-deoxypolysaccharides are highly nucleophilic and could be attacked by aldehydes to form the imine group at mild reaction conditions [34,35,36]. In our previous work, we reported the synthesis of 6-amino-6-deoxyinulin, which was conducted by the reaction between iminophosphorane intermediates of inulin and water [37]. On this basis, we have chosen to explore an experimental route which uses iminophosphorane intermediates of inulin as a substrate for the construction of the imine structure on inulin. Many articles have reported the influences of halogens on antifungal activity [38,39,40]. As a result, a series of aromatic aldehydes containing halogens were selected as the reactant. We expected a distinct improvement of antifungal activity of the obtained derivatives, which would provide a novel direction for the development of macromolecular fungicide. 

## 2. Experimental

### 2.1. Materials

Inulin (from chicory; average degree of polymerization was 20) was purchased from Haoyuan Biological Technology Co., Ltd., Xi’an, China. Triphenylphosphine (TPP, 95%); *N*-bromosuccinimide (NBS, 99%); cuprous iodide (99.5%); and all of the aromatic aldehydes such as benzaldehyde (98%), 2-fluorobenzaldehyde (98%), o-chlorobenzaldehyde (97%), 2-bromobenzaldehyde (98%), p-chlorobenzaldehyde (98%), and 3,4-dichlorobenzaldehyde (97%) were purchased from Sigma-Aldrich Chemical Co., Ltd., Shanghai, China. Other reagents such as *N,N*-dimethylformamide (DMF, 99.5%), acetic anhydride (98.5%), pyridine (99%), acetone (99.5%), diethyl ether (99.5%), triethylamine (99%), tetrahydrofuran (THF, 99%), and absolute ethyl alcohol (99.7%) were purchased from Sinopharm Chemical Reagent Co., Ltd., Shanghai, China. Potato dextrose agar (PDA) was purchased from Beijing Land Bridge Technology Co., Ltd., Beijing, China. All the reagents were used without other purification.

### 2.2. Analytical Methods

#### 2.2.1. Fourier Transform Infrared (FTIR) Spectroscopy 

The infrared spectra of inulin and inulin derivatives were measured on a Jasco-4100 infrared spectrometer (provided by JASCO Co., Ltd., Shanghai, China). The samples were measured with a KBr disk at room temperature (RT). The data were recorded in the range of 4000–400 cm^−1^.

#### 2.2.2. Nuclear Magnetic Resonance (NMR) Spectroscopy

The NMR spectra (1H NMR and 13C NMR) of inulin and inulin derivatives were measured on a Bruker AVIII 500 spectrometer (provided by Bruker Tech. and Serv. Co., Ltd., Beijing, China). Chemical shift values were recorded in the range of 0–10 ppm (1H NMR spectra) and 0–180 ppm (13C NMR spectra) at RT.

### 2.3. Synthesis 

**Scheme 1:**
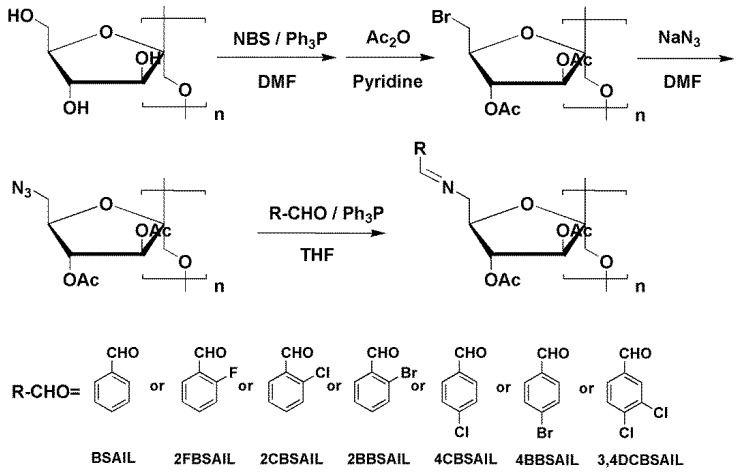
Synthetic pathway of inulin derivatives.

The synthesis routes of inulin derivatives are shown in Scheme 1. The synthesis of 6-azido-6-deoxy-3,4-di-O-acetyl inulin (AAIL) was performed on the basis of our previous methods with appropriate modifications [37]. The specific experimental steps are described below.

#### 2.3.1. Synthesis of 6-bromo-6-deoxy-3,4-di-O-acetyl Inulin

In 100 mL DMF, 30 mmol inulin was dissolved in a 250 mL flask. After that, 90 mmol NBS was added to the flask and stirred to clarification with a magnetic stirrer. Subsequently, the flask was placed in an ice bath until the temperature dropped to 0 °C. At that time, 90 mmol TPP was added to the flask within 30 min. After all the reagents were added, the solution was reacted at 80 °C for 3 h under argon atmosphere. After that, the solution was poured into excess acetone and the precipitates were separated by centrifugation and washed with acetone three times. Then, the obtained precipitates were dissolved in 100 mL pyridine and added to 15 mL acetic anhydride. The solution was stirred overnight at room temperature under argon atmosphere. Eventually, the solution was poured into excess ice water and the precipitates were separated by centrifugation and washed carefully with deionized water three times. The 6-bromo-6-deoxy-3,4-di-O-acetyl inulin was freeze-dried at −53 °C in vacuum. Yield: 87%; ^13^C NMR/DMSO-d6: *δ* 170 (C=O–acetyl), *δ* 33 (C-6-Br), *δ* 20 (CH_3_–acetyl), *δ* 60–103 (furanose rings); FTIR: *v* 3451 (OH), *v* 1735 (C=O of acetyl), *v* 661 (C-6-Br).

#### 2.3.2. Synthesis of AAIL

In a 250 mL flask, a mixture of 6-bromo-6-deoxy-3,4-di-O-acetyl inulin (10 mmol) and sodium azide (30 mmol) in 100 mL DMF was stirred at 80 °C for 3 h under argon atmosphere. After that, the solution was precipitated into excess ice water and the precipitates were separated by centrifugation. Then, the crude products were washed with deionized water and ethyl alcohol several times. AAIL was obtained by freeze-drying at −53 °C in vacuum. Yield: 68%; ^13^C NMR/DMSO-d6: *δ* 170 (C=O–acetyl), *δ* 52 (C–6–N), *δ* 20 (CH_3_–acetyl), *δ* 60–103 (furanose rings); FTIR: *v* 3451 (OH), *v* 210*7* (C–6–azido), *v* 1734 (C=O of acetyl). 

#### 2.3.3. Synthesis of Inulin Derivatives 

In a 25 mL flask, 2 mmol AAIL, 6 mmol triphenylphosphine, and 60 mmol aromatic aldehydes were mixed and dissolved in 15 mL THF. The reaction was carried out with magnetic stirring at 50 °C. The reaction process was monitored by infrared spectroscopy. After the reaction was completed, the solution was poured into excess diethyl ether and the precipitates were separated by suction filtration and washed with diethyl ether and acetone three times. The eventual products were obtained by freeze-drying at −53 °C in vacuum after further purification in a Soxhlet apparatus with acetone for 48 h.

6-amino-(*N*-benzylidene)-6-deoxy-3,4-di-O-acetyl inulin (**BSAIL**): Yield: 64%; DS_imine_ 0.34; ^13^C NMR/DMSO-d6: *δ* 170 (C=O–acetyl), *δ* 162 (C-7=N), *δ* 128–136 (aromatic carbons), *δ* 21 (CH_3_–acetyl); FTIR: *v* 1742 (C=O of acetyl), *v* 1670 (imine), *v* 3061, 1595, 1540, 694, 757 (benzene ring).

6-amino-(*N*-2-fluorobenzylidene)-6-deoxy-3,4-di-O-acetyl inulin (**2FBSAIL**): Yield: 68%; DS_imine_ 0.53; ^13^C NMR/DMSO-d6: *δ* 170 (C=O–acetyl), *δ* 164 (C-7=N), *δ* 162 (C-9-F), *δ* 116–132 (C-8, 10-13), *δ* 21 (CH_3_–acetyl); FTIR: *v* 1739 (C=O of acetyl), *v* 1670 (imine), *v* 3060, 1582, 1530, 1486, 1455, 764 (benzene ring).

6-amino-(*N*-2-chlorobenzylidene)-6-deoxy-3,4-di-O-acetyl inulin (**2CBSAIL**): Yield: 75%; DS_imine_ 0.94; ^13^C NMR/DMSO-d6: *δ* 170 (C=O–acetyl), *δ* 162 (C-7=N), *δ* 129–135 (aromatic carbons), *δ* 21 (CH_3_–acetyl); FTIR: *v* 1740 (C=O of acetyl), *v* 1664 (imine), *v* 3062, 1592, 756, 692 (benzene ring).

6-amino-(*N*-2-bromobenzylidene)-6-deoxy-3,4-di-O-acetyl inulin (**2BBSAIL**): Yield: 82%; DS_imine_ 0.71; ^13^C NMR/DMSO-d6: *δ* 170 (C=O–acetyl), *δ* 162 (C-7=N), *δ* 124–134 (aromatic carbons), *δ* 21 (CH_3_–acetyl); FTIR: *v* 1740 (C=O of acetyl), *v* 1673 (imine), *v* 3062, 1589, 1529, 758 (benzene ring).

6-amino-(*N*-4-chlorobenzylidene)-6-deoxy-3,4-di-O-acetyl inulin (**4CBSAIL**): Yield: 79%; DS_imine_ 0.69; ^13^C NMR/DMSO-d6: *δ* 170 (C=O–acetyl), *δ* 162 (C-7=N), *δ* 129–139 (aromatic carbons), *δ* 21 (CH_3_–acetyl); FTIR: *v* 1735 (C=O of acetyl), *v* 1676 (imine), *v* 3062, 1596, 1490 (benzene ring).

6-amino-(*N*-4-bromobenzylidene)-6-deoxy-3,4-di-O-acetyl inulin (**4BBSAIL**): Yield: 71%; DS_imine_ 0.50; ^13^C NMR/DMSO-d6: *δ* 170 (C=O–acetyl), *δ* 162 (C-7=N), *δ* 121–135 (aromatic carbons), *δ* 21 (CH_3_–acetyl); FTIR: *v* 1741 (C=O of acetyl), *v* 1668 (imine), *v* 3062, 1592, 1485, 754 (benzene ring).

6-amino-(*N*-3,4-dichlorobenzylidene)-6-deoxy-3,4-di-O-acetyl inulin (**3,4DCBSAIL**): Yield: 88%; DS_imine_ 0.68; ^13^C NMR/DMSO-d6: *δ* 170 (C=O–acetyl), *δ* 162 (C-7=N), *δ* 128–136 (aromatic carbons), *δ* 21 (CH_3_–acetyl); FTIR: *v* 1739 (C=O of acetyl), *v* 1673 (imine), *v* 3062, 1592, 755, 727 (benzene ring).

### 2.4. Antifungal Assay

An antifungal assay was performed according to reports in the literature and minor modifications were necessary [25,28]. In brief, each sample was dispersed in distilled water and then mixed with sterilized PDA to make a series of final concentrations at 0.1, 0.5, and 1.0 mg/mL. After the mixture was transferred to a plate and cooled to solid, a piece of PDA (diameter of 5 mm) containing the test fungi was placed in the middle of the test plate and the mycelium was incubated at 27 °C and 60% humidity until the mycelium of fungi reached the edges of the control plate (without the presence of sample). At last, the inhibitory index was calculated as follows (Equation (1)):

Inhibitory index (%) = (1 − *D*_a_/*D*_b_) × 100 (1)

where *D*_a_ is the diameter of the growth zone in the test plates and *D*_b_ is the diameter of the growth zone in the control plates. All tests were repeated three times and the results were calculated as the mean.

### 2.5. Statistical Analysis

All the data related to the antifungal activity are displayed as mean ± standard deviation (SD, *n* = 3) for triplicates. Significant difference analysis was determined using Scheffé’s multiple range test. The significant differences were defined at *p* < 0.05.

## 3. Results and Discussion

### 3.1. Structure of the Inulin Derivatives

**Figure 1:**
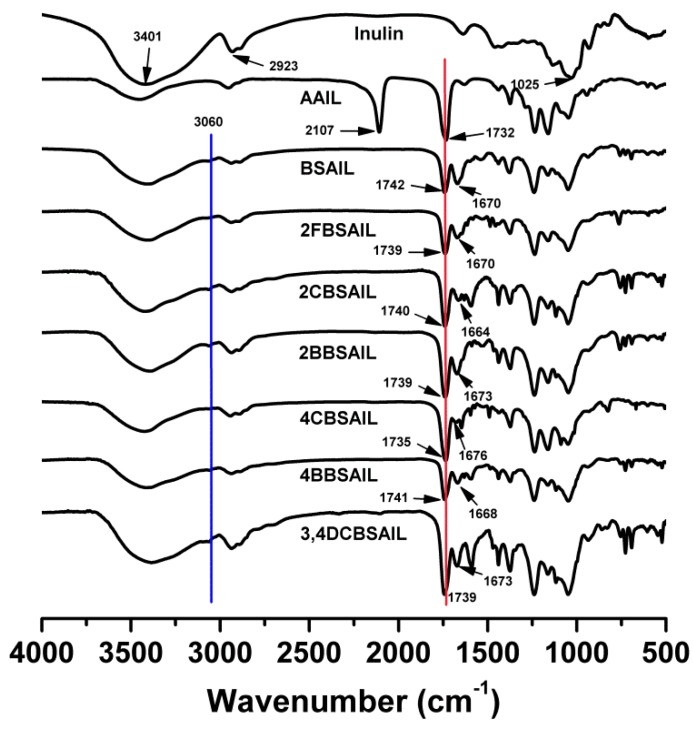
FTIR spectra of inulin, 6-azido-6-deoxy-3,4-di-O-acetyl inulin (AAIL), and inulin derivatives.

**Figure 2:**
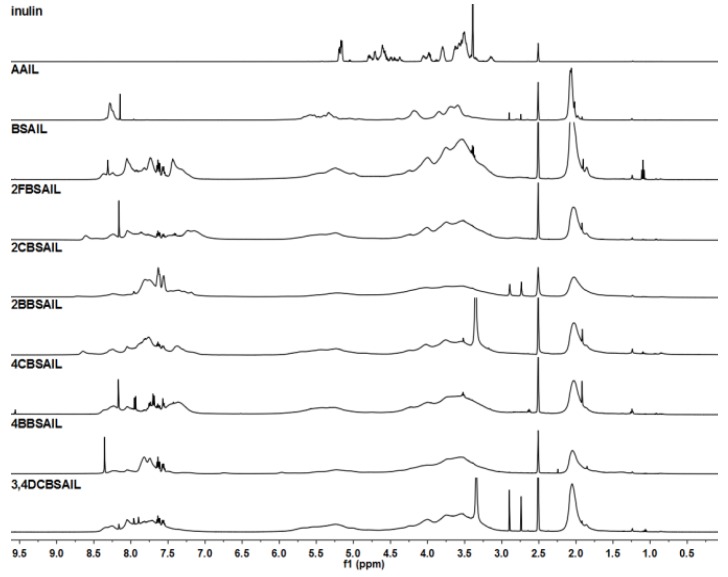
^1^H NMR spectra of inulin, AAIL, and inulin derivatives, recorded in DMSO-d6.

**Figure 3:**
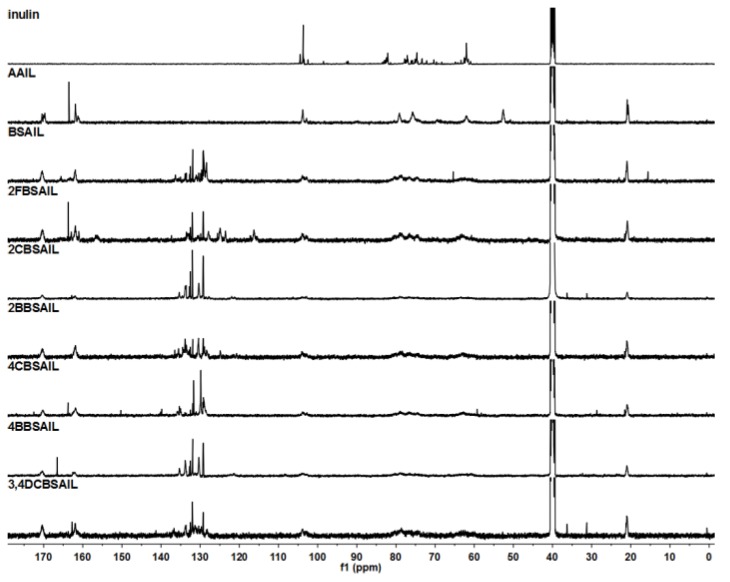
^13^C NMR spectra of inulin, AAIL, and inulin derivatives, recorded in DMSO-d6.

The structures of inulin and inulin derivatives were characterized by FTIR (Figure 1), ^1^H NMR (Figure 2), and ^13^C NMR (Figure 3) spectroscopy. As shown in Figure 1, the characteristic absorption peaks of saccharide were located at 3401 (O–H stretching), 2923 (–CH_2_ stretching), and 1025 cm^−1^ (C–O–C bending) [37]. In the IR spectrum of AAIL, there were distinct peaks at 2107 and 1734 cm^−1^ that belonged to the azido and acetyl, respectively [37]. Regarding inulin derivatives, compared with AAIL, the absorption of acetyl (1734 cm^−1^) still existed, but the absorption of azido (2107 cm^−1^) disappeared completely. Instead, there were new peaks at about 1670 cm^−1^, which belonged to the imine. Moreover, there were no absorptions of aromatic aldehydes (1700 cm^−1^) found in inulin derivatives, which indicated that these derivatives were well purified [35,41]. Besides, at 3060 and 1450–1600 cm^−1^, there appeared the characteristic absorption of the benzene ring. At the fingerprint region, absorptions at 754, 727, and 693 cm^−1^ were also evidence of the benzene conjunction [35].

^1^H NMR (Figure 2) and ^13^C NMR (Figure 3) were useful for further characterizing the structure of inulin, AAIL, BSAIL, 2FBSAIL, 2CBSAIL, 2BBSAIL, 4CBSAIL, 4BBSAIL, and 3,4DCBSAIL. The signals of the inulin skeleton, including the anhydroglucose unit, appeared at δ 3.00–5.40 ppm in ^1^H NMR and δ 60–103 ppm in ^13^C NMR [37], which always existed for every inulin derivative. After azide substitution, for the ^1^H NMR of AAIL compared to inulin, new signals appearing at δ 3.69 and δ 2.03 ppm were assigned to the H–C_6_–N and the hydrogen of the acetyl (Ac), respectively. Similarly, for the ^13^C NMR of AAIL, signals at δ 52.58 and δ 170.28 ppm were attributed to the C_6_-N and C=O of the acetyl (Ac), respectively. After further reaction, in the ^13^C NMR spectrum of inulin derivatives, the signals of C_6_-N moved to δ 63 ppm. Additionally, signals at about δ 164 ppm were assigned to the N=C_7_. Meanwhile, new chemical shifts appearing in the range of δ 120–140 ppm contributed to the aromatic carbons of the benzene ring. In the ^1^H NMR spectrum of inulin and inulin derivatives, signals appearing at about δ 8.05 ppm were assigned to the H–C_7_=N. Signals in the range of 7.00–8.60 ppm were assigned to the aromatic hydrogens of the benzene ring [35].

All of the abovementioned data proved the successful preparation of inulin derivatives.

### 3.2. Degree of Substitution (DS)

**Figure 4:**
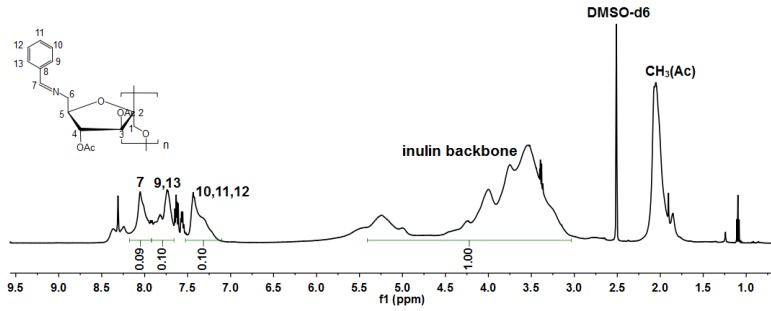
^1^H NMR spectrum of 6-amino-(*N*-benzylidene)-6-deoxy-3,4-di-O-acetyl inulin (BSAIL) and its integral values of hydrogen protons.

The DS for inulin derivatives was calculated by the ratio of the integral values of protons H_7-13_ to that of inulin backbone protons (H_1-6/6′_) [35,42,43,44]. For example, the DS_imine_ value of BSAIL was calculated according to Equation (2) by ^1^H NMR (Figure 4). The results are summarized in Table 1.

(2) $${\mathrm{DS}}_{\mathrm{imine}}=\frac{7\times I_{H,\mathrm{aromatic}+H,7}}{6\times I_{H,\mathrm{AGU}}}$$

where I represents the integral values, H,aromatic is the aromatic protons, H,7 is the proton of -CH=N, and H,AGU is the inulin backbone protons. The coefficient 7 means the amount of hydrogen protons on the inulin backbone, and the coefficient 6 means the amount of hydrogen protons on the benzene ring and imine.

### 3.3. Solubility and Antifungal Activity

**Figure 5:**
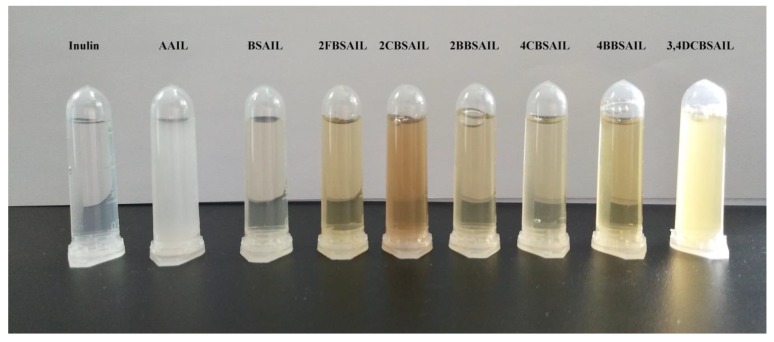
The water solubility of inulin, AAIL, and inulin derivatives at 1.0 mg/ mL, room temperature.

Figure 5 shows the aqueous solutions of samples at 1.0 mg/mL, room temperature. Except AAIL and 3,4DCBSAIL, all samples showed favorable water solubility and their solutions were prepared at a concentration of 0.1–1.0 mg/mL. For AAIL and 3,4DCBSAIL, considering that they are homodisperse in deionized water, their antifungal activity was tested together with other samples.

**Figure 6:**
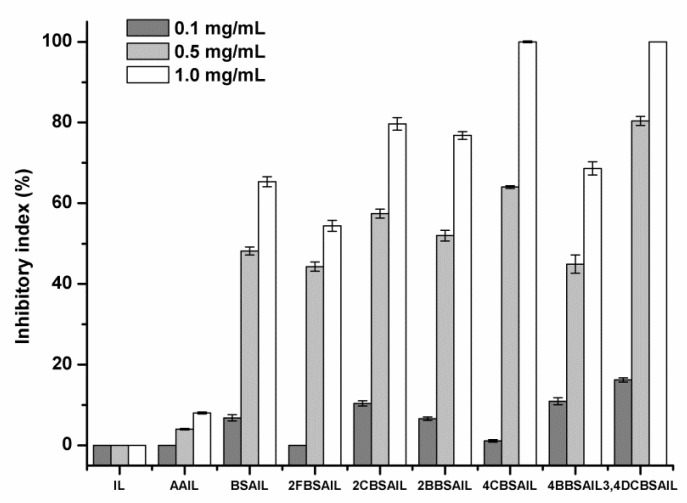
The antifungal activity of inulin, AAIL, and inulin derivatives against *Botrytis cinerea*.

**Figure 7:**
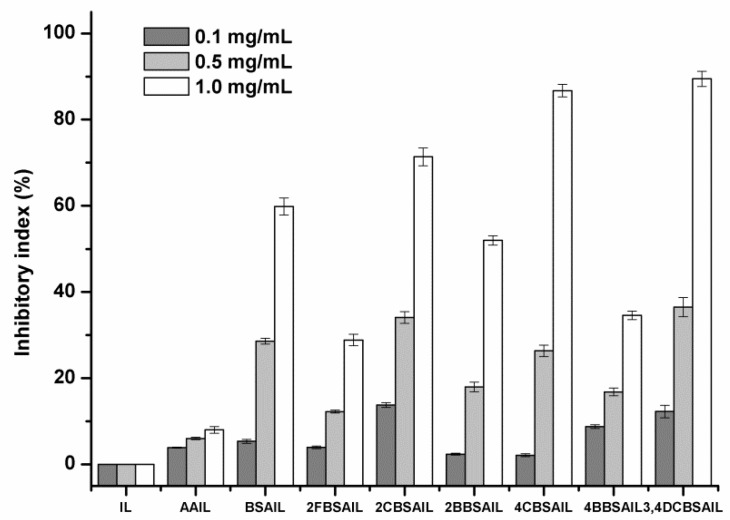
The antifungal activity of inulin, AAIL, and inulin derivatives against *Fusarium oxysporum* f. sp. niveum.

**Figure 8:**
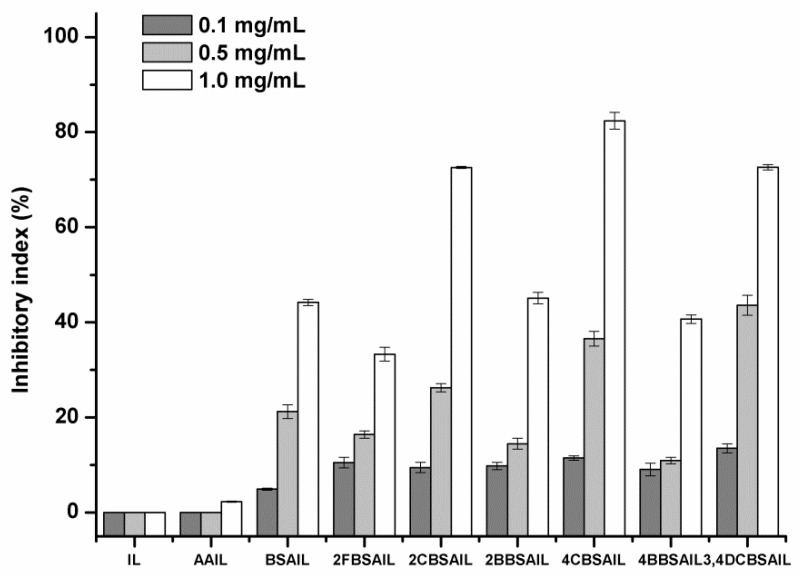
The antifungal activity of inulin, AAIL, and inulin derivatives against *Phomopsis asparagi*.

**Figure 9:**
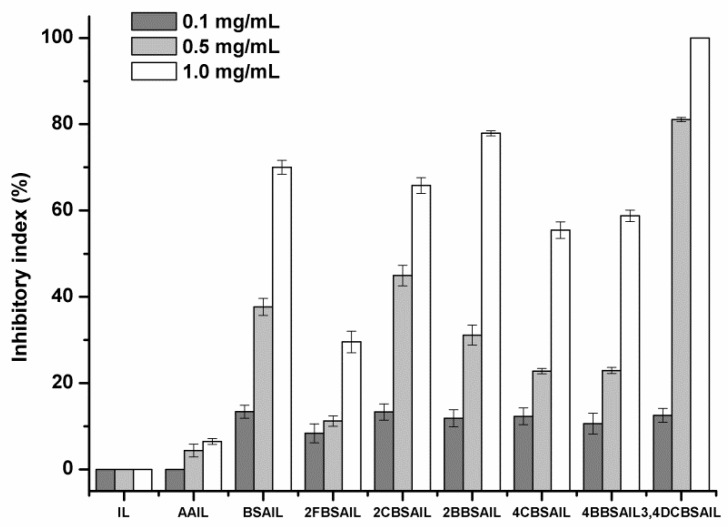
The antifungal activity of inulin, AAIL, and inulin derivatives against *Fusarium oxysporum* f. sp. cucumerium Owen.

Due to the pressure of environmental pollution, as well as the rising incidence of drug-resistant pathogens, antifungal polymers are part of a novel approach to developing antifungal agents that have the advantages of high efficiency, broad spectrum, low toxicity, slow release, and so forth. Annually, economic losses for farmers caused by pathogenic fungi are tremendous. As a result, four kinds of plant pathogens, namely, *Botrytis cinerea, Fusarium oxysporum* f. sp. cucumerium Owen, *Fusarium oxysporum* f. sp. niveum, and *Phomopsis asparagi*, were selected to evaluate the antifungal activity of the synthesized inulin derivatives by the mycelium growth rate method. Each sample was tested at 0.1, 0.5, and 1.0 mg/mL. The results are shown in Figure 6, Figure 7, Figure 8 and Figure 9.

As shown in Figure 6, Figure 7, Figure 8 and Figure 9, inulin exhibited no inhibitory effect against the test fungi due to the lack of a functional group. AAIL showed negligible antifungal activity, as azide grafting had no effect on antifungal action. Regarding inulin derivatives, at the test concentrations, all of them exhibited distinct antifungal activity compared with inulin against the four test fungi, and the inhibitory indices increased with increasing concentrations. 

Figure 6 shows the antifungal activity of inulin, AAIL, and inulin derivatives against *B. cinerea*. For the seven kinds of inulin derivatives (BSAIL, 2FBSAIL, 2CBSAIL, 2BBSAIL, 4CBSAIL, 4BBSAIL, and 3,4DCBSAIL), their inhibitory rates ranged from 54% to 100% at 1.0 mg/mL. The inhibitory rate of BSAIL was 48% at 0.5 mg/mL, and the value reached 65% at 1.0 mg/mL. In other words, the introduction of Schiff bases to inulin helped confer to it high antifungal activity. When the Schiff bases were added with halogen substituent groups, the antifungal activity of inulin derivatives was subsequently affected. The inhibitory rates of the inulin derivatives were in the following order: 3,4DCBSAIL > 4CBSAIL > 2CBSAIL > 2BBSAIL > 4BBSAIL > BSAIL > 2FBSAIL. The inhibitory rates of 3,4DCBSAIL, 4CBSAIL, 2CBSAIL, 2BBSAIL, and 4BBSAIL were greater than that of BSAIL, which indicates that chlorine and bromine could help to further improve the antifungal activity of inulin on the basis of the Schiff bases. The inhibitory rates of 3,4DCBSAIL and 4CBSAIL were both 100% at 1.0 mg/mL, and at the same concentration, the inhibitory rates of 2CBSAIL, 2BBSAIL, and 4BBSAIL were 80%, 77%, and 69%, respectively. On the contrary, the introduction of fluorine decreased the antifungal activity of inulin compared with BSAIL, as the inhibitory rate of 2FBSAIL was 54% at 1.0 mg/mL. By analyzing the inhibitory indices of 2CBSAIL and 4CBSAIL, we found that chlorine substituted at the para-position of benzene was more effective than that substituted at the ortho-position. However, this result was in contrast to that of 2BBSAIL and 4BBSAIL, and the inhibitory rate of 2BBSAIL was higher than 4BBSAIL. Moreover, it was found that the inhibitory rate of 3,4DCBSAIL was larger than 2CBSAIL and 4CBSAIL, which means that the antifungal activity of the inulin derivatives with double chlorine was stronger than that with single chlorine against *B. cinerea*. 

Figure 7 and Figure 8 shows the antifungal activity of samples against *F. oxysporum* f. sp. niveum and *P. asparagi*, respectively. The results were similar to those listed above in some respects. Firstly, the Schiff bases with different kinds of substituent groups (halogens) had different effects on the antifungal activity of inulin derivatives. Chlorine was superior to bromine, and bromine was superior to fluorine. Secondly, for the same halogen, chlorine substituted at the para-position of benzene was more efficient at improving the antifungal activity of inulin derivatives than that substituted at the ortho-position, but the regularity was the opposite for fluorine. Moreover, 3,4DCBSAIL always exhibited rather strong antifungal activity. Its inhibitory indices against *F. oxysporum* f. sp. niveum and *P. asparagi* were 89% and 73%, respectively.

Figure 9 shows the antifungal activity of samples against *F. oxysporum* f. sp. cucumerium Owen. All the inulin derivatives exhibited significant antifungal activity and the inhibitory rate of 3,4DCBSAIL was 100% at 1.0 mg/mL. These results further confirm the advantage of grafting Schiff bases onto inulin to impart high antifungal activity to inulin. 

Among the four test fungi, *B. cinerea* exhibited the most sensitivity to the inulin derivatives, and the inhibitory indices of the inulin derivatives were all above 50% at 1.0 mg/mL, which was related to the synergistic role of halogens. 3,4DCBSAIL exhibited the strongest antifungal activity for both strains of *F. oxysporum*. However, *F. oxysporum* f. sp. niveum was more sensitive to the derivatives containing chlorine. Differently, *F. oxysporum* f. sp. cucumerium Owen was easier to be restrained by the derivatives containing bromine.

## 4. Conclusions

In this study, seven kinds of inulin derivatives modified by Schiff bases were successfully synthesized. Their structure characteristics were confirmed by FTIR, ^1^H NMR, and ^13^C NMR. The antifungal activity of these derivatives against four kinds of plant pathogens, namely, *B. cinerea, F. oxysporum* f. sp. cucumerium Owen, *F. oxysporum* f. sp. niveum, and *P. asparagi*, was studied by the mycelium growth rate method in vitro. Firstly, the results confirmed the feasibility of modifying inulin with Schiff bases to confer high antifungal activity to inulin. Then, we found that the type, the position, and the number of substituent groups (halogens) on Schiff bases had an effect on the derivatives’ antifungal activity. The halogens could have a synergistic effect, as they exhibited a variety of biological activities, including antifungal activity and electron-withdrawing capacity. In general, chlorine was more helpful to further improve the derivatives’ antifungal activity on the basis of Schiff bases. More chlorine resulted in stronger antifungal activity. The same derivative had different inhibitory effects on different fungi. Many fungicides containing halogens have pronounced toxicities and their residues in the environment have already caused serious environmental problems. When these groups are grafted onto inulin, they could be released slowly, which would largely resolve the environmental issue. Further studies will be carried out to test this hypothesis.

This paper provides a practical strategy to develop novel inulin derivatives bearing Schiff bases with high antifungal activity. The products described in this paper have great potential as alternatives to some harmful pesticides used for plant disease control.

## Figures and Tables

**Table 1:** The yield and the degree of substitution of inulin derivatives.

Compound	Yield	Degree of Substitution
Inulin	/	/
BSAIL	64%	0.34
2FBSAIL	68%	0.53
2CBSAIL	75%	0.94
2BBSAIL	82%	0.71
4CBSAIL	79%	0.69
4BBSAIL	71%	0.50
3,4DCBSAIL	88%	0.68
